# Postpartum modern contraceptive use in northern Ethiopia: prevalence and associated factors - methodological issues in this cross-sectional study

**DOI:** 10.4178/epih.e2017019

**Published:** 2017-05-10

**Authors:** Kamyar Mansori, Shiva Mansouri Hanis, Fatemeh Khosravi Shadmani

**Affiliations:** 1 Social Development and Health Promotion Research Center, Gonabad University of Medical Sciences, Gonabad, Iran; 2 Department of Epidemiology, School of Public Health, Iran University of Medical Sciences, Tehran, Iran; 3 School of Public Health, Dezful University of Medical Sciences, Dezful, Iran; 4 Department of Epidemiology, School of Public Health, Shahid Beheshti University of Medical Sciences, Tehran, Iran

Dear Editor,

We read the paper entitled ‘Postpartum modern contraceptive use in northern Ethiopia: prevalence and associated factors,’ written by Abraha et al. [1], which was published in *Epidemiology and Health* in March 2017. The aim of the study was to assess postpartum modern contraceptive use and associated factors among postpartum women in northern Ethiopia. A multivariable logistic regression model showed that the following independent variables were the most important determinants of postpartum modern conception use in the town of Aksum: maternal educational level (secondary and tertiary education level), family planning counseling during pregnancy and during prenatal and postnatal care, having postnatal care, resuming sexual activity, menses returning after birth, and experiencing problems with previous contraceptive use [1]. However, although this research was valuable and the results are interesting, some methodological issues should be considered relating to this cross-sectional study.

Regardless of the results obtained from the model, it should be explained that accurate predictors or determinants of a dependent variable cannot be reliably identified by a cross-sectional study because predictors must be identified based on cohort studies [2,3]. In other words, predictive or casual inferences cannot be made from cross-sectional studies because of the associations between variables measured at the same time point in such studies. Without the temporality assumption (the dependent variable must occur after the independent variable) there is no way of determining whether a factor is a risk factor, is predictive/causal, or is a consequence of the outcome [4]. Therefore, longitudinal studies are essential for developing assumptions to be used in clinical prediction models, whereas in this study [1], a cross-sectional study was used to identify the independent predictors of postpartum modern contraceptive use. Therefore, it is essential to interpret the results of this study in light of the above explanation.
